# Caretaker Score Reliability for Personality Assessment of Bottlenose Dolphin (*Tursiops truncatus*)

**DOI:** 10.3390/ani11072073

**Published:** 2021-07-12

**Authors:** Marina Salas, Amanda Fernández-Fontelo, Eva Martínez-Nevado, Jesús Fernández-Morán, Agustín López-Goya, Xavier Manteca

**Affiliations:** 1 Facultat de Veterinària, Universitat Autònoma de Barcelona, 08193 Bellaterra, Spain; xavier.manteca@uab.cat; 2 Antwerp Zoo Centre for Research and Conservation (CRC), Royal Zoological Society of Antwerp (KMDA), 2018 Antwerp, Belgium; 3 Chair of Statistics, School of Business and Economics, Humboldt-Universität zu Berlin, 10099 Berlin, Germany; fernanda@hu-berlin.de; 4 Zoo Aquarium de Madrid, Casa de Campo, s/n, 28011 Madrid, Spain; emartinez@grpr.com (E.M.-N.); jfernandezm@grpr.com (J.F.-M.); Algoya@grpr.com (A.L.-G.)

**Keywords:** animal keeper, animal personality, behaviour, captivity, dolphinarium, intra-rater dependence, temperament, welfare, zoo

## Abstract

**Simple Summary:**

The assessment of animals’ personalities can help manage decisions concerning zoo animals more appropriately; for example, a proper personality evaluation helps create stable social groups or increase the chances of breeding success if compatible breeding pairs are chosen. In zoos, the animals’ personality is often evaluated by the caretakers due to their familiarity with the animals and their behaviours. In this study, we aimed to evaluate how reliable caretakers’ ratings are when assessing dolphins’ personalities. With this aim in mind, we asked 24 caretakers to score a variety of personality traits of bottlenose dolphins under their care through a questionnaire in two periods. Our findings showed fair to good degrees of agreement within scores of the same rater and across raters within the same centre. We were also able to identify which raters and centres showed significant score mean differences systematically. We believe the study of raters’ outcomes reliability is crucial to make appropriate management decisions based on the animals’ personalities.

**Abstract:**

The evaluation of zoo animals’ personalities can likely lead to a range of benefits, including improving breeding success, creating stable social groups, and designing and developing environmental enrichment programmes. The goal of this study was to use caretakers scores to evaluate personality in bottlenose dolphins and to assess the reliability of scores within each rater and among raters from each centre. To this end, 24 caretakers from 3 countries (Spain, France, and Argentina), including a total of 5 dolphinariums and 6 groups of dolphins, used a questionnaire based on the Five-Factor Model of Personality to score bottlenose dolphins on a number of personality traits in three different contexts. Each caretaker evaluated the animals under their care twice, ensuring that raters did not share thoughts nor impressions with other raters. Our findings showed a good degree of agreement between each rater’s scores and a fair degree of agreement among scores of raters from the same centre. We also identified which raters and centres had significant mean score differences and detected that 4 out of 24 raters from two different centres showed such differences systematically. The evaluation of raters’ reliability and the identification of particular inconsistent raters and centres is critical to make more appropriate and realistic management decisions that, in turn, directly impact animals’ welfare.

## 1. Introduction

‘Personality’ is defined as ‘individual differences in behaviour that are thought to be stable across time and situations’ [1] (p. 654). Personality stems from the interaction between genetic and environmental factors, and it is widely accepted that individual animals have different personalities, which are likely to affect their welfare in captivity [2,3,4,5]. Among the benefits, it has been suggested that knowing certain personality traits can help improve reproductive success, such as identifying compatible breeding pairs [6,7]. In addition, the personality of individuals within a social group can impact social compatibility and stability [8]. Therefore, the assessment of personality can be used to decide which animals should be housed together when planning the introduction of new members [9]. The study of personality is also important for daily husbandry, i.e., each individual’s response to environmental enrichment programmes will depend on its personality [10]. For example, the same stimulus can have a positive impact on increasing exploratory behaviour in an animal which is generally bold, but a negative effect on a fearful individual [11]. Moreover, how visitors perceive animals in zoos are likely to be affected by animal personalities, and this can, in turn, affect the effectiveness of zoo education initiatives [2].

Two methods for assessing personality in animals have been described in the literature in past years [1,2,9,12]. One uses animal observations, including the frequency, duration, or intensity of a variety of behaviours that characterise the animals’ personality [13]; the second, which is the most commonly used for evaluating personality in zoo animals [9,14,15,16,17], assesses personality traits with observer ratings. Raters (or observers) are expected to have sufficient experience with each individual so that their overall impression of each animal reflects the animal’s personality fairly [2]. Animal caretakers and trainers are familiar with the animals in their care and have seen how they respond to various situations and stimuli. They can rate animal personality traits in a reliable manner based on their familiarity with and long-term observations of the animals in their care, as they can evaluate how consistent behavioural traits are and how these are manifested [2,9].

The assessment of personality has to be reliable and valid [1,18]. The agreement among the raters in their scoring assessments or behavioural observations is measured by testing the intra-rater or intra-observer reliability [18,19]. Previous studies on the assessment of personality in different species have shown strong or intense intra-rater reliability, i.e., strong or intense degrees of agreement between scores for the same individual given by different raters, thus evident signs of consistency between raters. A review presented by Tetley and O’Hara [9] concluded that 93% of the personality studies using observer raters examined the intra-rater reliability. In addition, they concluded that all reviewed authors found moderate to strong intra-rater reliabilities in their personality studies, indicating that ‘raters are able to reach a statistically confirmed agreement on the expression of traits in individual animals’ (p. 469).

There are personality questionnaires based on the Five-Factor Model of Personality, a model that has been used to study human personality and also other animals’ personalities, especially primates. The five factors are: (1) Openness to experience, (2) Conscientiousness, (3) Extraversion, (4) Agreeableness, and (5) Neuroticism. The personality questionnaires consist of a list of adjectives, and raters are asked to score each animal on these adjectives. To check the reliability of primates personality assessments that used the Five-Factor Model of Personality, the intra-rater reliability was calculated and found to be high and without significant differences between ratings in chimpanzees [20,21] and orangutans [22], among others.

The Five-Factor Model of Personality has also been considered to study bottlenose dolphins’ (*Tursiops truncatus*) personalities [23,24]. Personality characteristics in this have been considered to be influenced by context (interactions with the physical environment, humans, and other dolphins), and therefore it would be important to consider the context when assessing a dolphin’s personality [24].

Dolphins’ personalities have been previously investigated on a few occasions, in both the wild and in captivity. For example, Díaz López [25] studied the reaction of 24 wild bottlenose dolphins towards two novel and threatening situations. His method considered the presence of an underwater observer wearing snorkelling gear and used an acoustic harassment device to measure the distance between the novel objects and each dolphin, allowing the observer to assess how bold or shy each individual was. In addition, the author discussed how personality could influence, and thus structure, social networks in a population of wild bottlenose dolphins. In the same line, Highfill and Kuczaj [23] looked into the personality of 15 captive dolphins in two different locations (MarineLife Oceanarium in Gulfport, Mississippi, USA, and Atlantis Resort in the Bahamas) before Hurricane Katrina and 15 months later. The authors considered a rater-type approach to evaluate dolphins’ personalities on a seven-point scale and distributed the raters into two different groups. One group of raters at the location before the hurricane occurred and a second group at the new location after the hurricane occurred. For both groups, only raters who had a minimum of 1 year of experience with each dolphin participated in the study. The authors finally concluded that dolphins have different personalities, which are relatively stable over time and across situations. Another example can be found in Birgersson [26], who considered a different approach studying a group of eight captive dolphins with the Five-Factor model and focal observations. The results showed that the animals had distinct personality traits and similarities in these five factors and that the dolphins preferred the company of some individuals over others.

To summarise, these studies employed two different methods for dolphins’ personality evaluations (i.e., behavioural observations and observer ratings) and demonstrated that dolphins have different personalities, which can be assessed both in captivity and in the wild. However, in the two studies described above, in which authors used behavioural observations [25,26], the evaluation of dolphins’ personalities was completed by just one researcher. On the other hand, in the study using observer ratings [23], there were two different groups of raters (i.e., different groups of raters with different individuals in each group) who assessed the dolphins’ personalities. In accordance with that, our current study aimed to investigate whether the use of caretakers’ scores to evaluate personality in captive bottlenose dolphins measures such a personality consistency. In particular, our goal was to investigate the consistency or reliability of scores within the rater and among the raters from each participant dolphinarium based on different personality traits (i.e., adjectives). However, our work differs from the works above [23,25,26] in that we used the same group of caretakers to evaluate the personality of the dolphins under their care twice. Note that our focus was not on the consistency of the scores over time, but on whether each rater scored each dolphin homogeneously for a given adjective in two independent tests conducted under equivalent conditions.

## 2. Materials and Methods

### 2.1. Personality Questionnaire

Our analysis was based on the scores collected from a questionnaire designed to assess personality traits in bottlenose dolphins. This questionnaire (elaborated in English) was based on the Five-Factor Model of Personality, adapted from Kuczaj et al. [24] and Highfill et al. [23], and included 25 adjectives described in further detail in Table 1. The questionnaire was divided into the following three sections: (1) interactions with the physical world: concerning how dolphins interact with their physical environment, including objects, (2) interactions with other dolphins: concerning how dolphins behave towards other dolphins, and (3) interactions with humans: concerning how dolphins behave towards humans. For each section, the caretaker scored each dolphin in terms of a list of adjectives. The adjectives were scored using a seven-point scale. For example, for the adjective ‘Not curious/Curious’ the rater had to choose among the following response choices: ‘extremely not curious’, ‘quite not curious’, ‘slightly not curious’, ‘neutral’, ‘slightly curious’, ‘quite curious’, ‘extremely curious’. There was also the option of responding ‘do not know’. Each questionnaire was answered anonymously.

### 2.2. Participants

We collaborated with five centres that housed dolphins from three different countries: Spain, France, and Argentina. In total, six groups of dolphins participated in this study. We asked 24 animal caretakers or trainers to rate the animals on the 24 different adjectives (described in Table 1) in each of the three sections using the questionnaires described above. Table 2 describes the distribution of each centre in terms of the number of scored dolphins by sex and the identification code (and total number) of caretakers. Only trainers or caretakers who had been for more than a year interacting with each to-be-scored dolphin filled in a personality questionnaire, as recommended by Kuczaj et al. [24] and Highfill and Kuczaj [23]. In addition, an important particularity of our study was that each caretaker scored each dolphin in their centre in two independent tests, at different times (the time between test 1 and test 2 for each caretaker was about 3–4 weeks), without checking the score given to each dolphin in test 1 when test 2 was conducted, and without discussing or conferring the answers with other raters [9]. The dolphins were scored between the end of 2017 and the beginning of 2018.

### 2.3. Statistical Analysis

We used a two-step procedure to assess scores’ reliability within each rater and between raters from the same centre, i.e., to estimate the degree of agreement between the scores for the same individual given by the same rater (tests 1 and 2), as well as the degree of agreement between the scores for the same individuals given by the raters within the same centre, and to identify particularly which centres and raters show significant mean score differences.

First, we estimated the intra-class correlation coefficient of the scores in tests 1 and 2 given by the same rater for the same dolphin, section, and adjective to evaluate the intra-rater reliability (i.e., the degree of agreement of scores of each rater for each animal in tests 1 and 2), and the intra-class correlation coefficients (for test 1 and test 2 separately) of the scores given by the raters within the same centre for the same dolphin, section, and adjective to evaluate the intra-centre reliability (i.e., the degree of agreement of scores in tests 1 and 2 separately of raters within each centre for the same dolphin). Second, we wanted to identify which raters and centres had significant differences, on average, in their scores for each of the sections and adjectives, considering further the possibility for interdependence across observations from the same dolphin, as this information may be especially helpful in spotting problems related to caretakers who do not know their animals well enough to assess their personalities. Therefore, note that we evaluated raters’ and centres’ reliabilities using an agreement measure, e.g., the intra-class correlation coefficient (see below), as well as by identifying raters and centres showing mean score differences between tests 1 and 2 systematically. Through this methodology, we not only estimated raters and centres agreement in their scores with an aggregate-type measure, i.e., a measure such as the intra-class correlation coefficient that gives aggregated information on the dependence of the outcomes of interest (e.g., scores) within a cluster, but we also identified potentially deviating raters and especially centres which most contributed to reducing the degree of agreement in our outcomes.

With the purpose of measuring the intra-rater reliability, we estimated a linear mixed model to regress the scores given by the raters (response variable) onto sex and age (explanatory variables) as fixed effects and centre, centre:rater (to ensure the nested structure of raters within centres), dolphin and section:adjective:dolphin (to ensure the nested structure of adjectives evaluated several times over the same dolphin in the three different sections of the questionnaire) as random effects in the intercept only. With such a model, we can decompose the variability of scores (response variable) into the variabilities due to the dolphin, centre, rater (considering its nested dependence with centres), adjective (considering its nested dependence with section and dolphin), as well as the residual variability (i.e., the variability of the scores not explained by the estimated model). Therefore, the intra-rater reliability can be evaluated with the intra-rater correlation coefficient as the quotient of the sum of variances due to dolphin, centre, centre:rater and section:adjective:dolphin by the total variability of the response variable, and can be interpreted as the correlation between the scores (i.e., two scores from tests 1 and 2) given by the same rater for the same dolphin, section and adjective. In addition, to estimate the intra-centre reliability, we used the same model as above, but stratifying our data set by test in such a way that we could compute the intra-centre correlation coefficients of scores in test 1 (and similarly and separately in test 2) for the raters within the same centre for the same dolphin, section and adjective. Thus, the intra-centre correlation coefficient for either test 1 or test 2 is estimated as the quotient of the sum of variances due to dolphin, centre and section:adjective:dolphin divided by the total variability of the scores.

Finally, to identify the centres and raters showing more statistically significant differences in their scores (on average), we wanted to compare the mean of scores between tests 1 and 2 given by the same rater for the same section and adjectives, as well as the mean of scores between tests 1 and 2 given by the same centre for the same section and adjective, accounting for the possibility of interdependence across the observations from the same dolphin. With such a purpose, we estimated another linear mixed model with sex, age, centre, rater, section, adjective and test (explanatory variables) as fixed effects, and dolphin as a random effect (i.e., scores for the same rater, centre and adjective are not necessarily independent, as those from the same dolphin may be highly correlated, thus accounting for interdependencies within each dolphin’s observations). On the basis of this model, we estimated and compared the corresponding mean of scores in tests 1 and 2 given by the same rater for the same section and adjective, as well as the mean of scores in tests 1 and 2 given by raters within the same centre for the same section and adjective. Additionally, we computed and compared such mean scores stratifying by sex as we were further interested in investigating whether these mean differences were independent of sex (i.e., whether or not an inconsistent rater showed systematically mean differences independent of the sex of the individuals) or on the contrary, if mean differences are found more frequently in females assessment compared to males assessment, or the other way around. 

We selected the above-described linear mixed model for evaluating both the intra-rater and intra-centre variability in such a way that we could decompose the variability of the scores into the variabilities due to dolphin, centre, rater, section and adjective and thus compute both the intra-rater and intra-centre correlation coefficients appropriately. In particular, we considered different combinations of such random effects, e.g., centre, rater, section, adjective and dolphin, independently as random effects in the intercept only and the intercept and slope, as well as different (and appropriate) nested structures among these random effects. We finally selected the above-described model, i.e., a model with the random effects described above, as this was the one, among our candidates, with the minimum Akaike Information Criterion (AIC). In addition, we chose the second linear mixed model to identify centres and raters with significant mean differences between their scores in tests 1 and 2 for a given section and adjective also based on the AIC. Note that we considered two different models here as the combined model, i.e., a model with random effects in dolphin, centre, centre:rater, and section:adjective:dolphin, and age, sex, centre, rater, section, adjective and test as fixed effects as well, was too complex and computationally unfeasible. 

Our analysis was performed with R 3.2.2. software and, particularly, the packages lme4, nlme, and lsmeans.

### 2.4. Ethical Approval

This study did not require ethical approval since it did not involve any interventions or handling of the animals.

## 3. Results

For the first part of the analysis (i.e., to evaluate both the intra-rater and intra-centre reliabilities), we obtained that the total variability of the scores (response variable) was 2.398 and could be decomposed into the variabilities due to the centre (0.038), centre:rater (0.130), section:adjective:dolphin (0.961), dolphin (0.344), and residual (0.924). Therefore, the intra-rater correlation coefficient is estimated as 0.615 (61.5%), indicating that the correlation between the scores for tests 1 and 2 given by the same rater on the same dolphin, for the same section and adjective, was good according to the scale proposed by Cicchetti [27]. In addition, to evaluate the degree of agreement between scores in test 1 given by the raters within the same centre for the same dolphin, section, and adjective, we obtained that the total variability of the scores in test 1 was 2.409 and could be decomposed into the variabilities due to the centre (0.025), section:adjective:dolphin (0.976), dolphin (0.367), and residual (1.041), and thus, the intra-centre correlation coefficient for test 1 is estimated as 0.568 (56.8%). In a similar way, the variability of scores in test 2 was 2.340 and could be decomposed into the variabilities due to the centre (0.115), section:adjective:dolphin (0.729), dolphin (0.313), and residual (1.183), and thus the intra-centre correlation coefficient for test 2 is estimated as 0.495 (49.5%). Again, in accordance with the scale proposed by Cicchetti [27], both intra-centre correlation coefficients for tests 1 and 2 are fair.

Second, to identify centres and raters showing inconsistencies in terms of differences in means between the scores in tests 1 and 2, we estimated a linear mixed model with sex, age, centre, rater, section, adjective, and test as fixed effects, and dolphin as a random effect in the intercept only. Table 3, Table 4 and Table 5 give the statistically significant mean differences between tests 1 and 2 according to the variables of interest, i.e., centre, rater, section, adjective, and test. In particular, Table 3 displays the significant mean differences between scores in tests 1 and 2 for each rater, section, and adjective, and thus, the raters who showed significant differences between their mean scores in tests 1 and 2. In particular, we found that raters 7, 8, 10, 12, 13, 16, 18, and 21 showed at least one significant difference between their mean scores in tests 1 and 2 for the same dolphin, section, and adjective. However, raters 7, 16, 18, and 21 only showed one discrepancy among their scores over the same sample of dolphins, section, and adjective. Therefore, we did not consider such raters systematically showing mean differences between scores in tests 1 and 2 over the same sample of dolphins, scores, and adjectives. However, raters 8, 10, 12, and 13 (4 raters out of 24) were considered to be inconsistent with their scores as they showed a relatively large number of differences between the mean of scores in tests 1 and 2 over the same sample of dolphins, sections, and adjectives. In particular, rater 13 (from centre D) showed the most number of differences in their mean scores between tests, particularly identifying significant mean score differences between tests in 8 activities (section and adjectives).

Table 4 shows the significant mean differences of scores in tests 1 and 2 for each centre, section, and adjective. The scores given to each adjective and section by the raters of centres A, B, E, and F did not show significant mean differences between tests 1 and 2. However, several incoherencies were found among the mean scores between tests 1 and 2 given by raters of centres C and D. This result agrees with the results above, as raters 8, 10, 12, and 13 (see Table 3) belong to these two centres.

The age of the dolphins was not statistically significant. However, the sex of the dolphins was relevant, and thus we considered investigating the differences given in Table 3 and Table 4 but stratifying by sex. In doing so, when studying the discrepancies of mean scores in tests 1 and 2 within the raters and among the raters of the same centre per section, adjective, and sex, we observed that the statistical differences described before for both within rater and among raters of the same centre still remain. Table 5 presents the statistically significant differences between mean scores in tests 1 and 2 for each rater, centre, section, adjective, and sex of the dolphins. Overall, we observed more statistically significant differences between mean scores in test 1 and test 2 (especially in sections 2 and 3) when assessing females rather than males.

It would appear that one of the most difficult adjectives to score was ‘Gentle’ (Table 5). We detected statistically significant differences between the average scores of test 1 and test 2 when assessing ‘Gentle’ in females in section 3 (average of 4.700 vs. 5.030, *p*-value = 0.047). Moreover, statistically significant differences were also detected between average scores of test 1 and test 2 when assessing ‘Gentle’ in males both in section 2 (average of 4.002 vs. 4.410, *p*-value = 0.047) and section 3 (average of 4.631 vs. 5.043, *p*-value = 0.044).

## 4. Discussion

Previous studies have confirmed that personality traits in bottlenose dolphins are consistent across time. Two approaches for personality evaluation have been considered in the literature, i.e., with behavioural observations or observers’ ratings. The work by Díaz López [25], for example, used behavioural observations to evaluate 24 wild bottlenose dolphins on several occasions, intending to assess specific personality traits towards two novel and threatening situations. Based on behavioural observations, Birgersson [26] analysed and compared behavioural data and the Five-Factor model in a group of eight dolphins in captivity, showing that the animals had distinct personality similarities and differences. However, these two studies used one researcher to conduct behavioural observations of the dolphins. Highfill and Kuczaj [23], on the other hand, studied the personality traits of 15 dolphins in two locations in captivity, before and after hurricane Katrina, with observer ratings. Yet, they used a different group of raters to score the animals in each of the locations.

To our knowledge, there have been no studies of dolphins’ personalities assessment in which the same group of raters evaluates the same sample of dolphins several times, even though this appears to be the most appropriate way for measuring intra-rater reliability. In accordance with that, the aim of our study was to investigate how reliable caretakers’ scores are for assessing the personality of captive bottlenose dolphins using raters scores collected in two independent tests, i.e., raters scored twice the same sample of animals using the questionnaire described above. We did not change the animals’ conditions in the two tests, as we were more interested in seeing whether the raters were consistent among themselves. In addition, we were also interested in whether the caretakers within each dolphinarium had the same perception of the personality of each dolphin. To this end, we collected scores on a sample of 24 caretakers who answered the personality questionnaire described above twice. Overall, we obtained rates on a total of 42 dolphins (separated into six social groups).

We used a two-step analysis for evaluating scores consistency for each rater and between raters within the same centre. First, we focused on assessing the degree of agreement between the scores within each rater (tests 1 and 2) for the same dolphin, section and adjective (intra-rater reliability) and the degree of agreement between the scores of raters within the same centre for the same dolphin, section, and adjective (intra-centre reliability). Both the intra-rater and intra-centre reliabilities are measured, respectively, with the intra-rater correlation coefficient and the intra-centre correlation coefficient; thus, in this first step, we used aggregated and correlation-type measures to summarise the dependence of the observations within the clusters of interest (i.e., within rater and within centres). In particular, our estimated intra-rater correlation coefficient was 0.615, indicating a good [27] intra-rater reliability. In addition, our estimated intra-centre correlation coefficients for tests 1 and 2 were 0.568 and 0.495, respectively, meaning that both the intra-centre reliability for tests 1 and 2 were fair [27]. These results agree with the ranges of intra-class correlation coefficients described in other studies for animals’ personality evaluations [9]. 

Second, we wanted to identify which raters and centres showed significant mean score differences from test 1 to test 2 systematically. We found this step to be particularly important as the identification of raters and centres showing discrepancies among their scores could indicate that they do not properly acknowledge their animals’ different personalities in the way they thought and that this, in turn, could have an impact on the animals’ welfare. It has previously been seen in the literature that the detection of certain personality traits can help identify compatible breeding pairs [6,7], detect which individuals can potentially impact social compatibility and stability within a group [8], or decide which animals should be held together when planning the introduction of new individuals [9]. In addition, changes in behaviour and temperament can be an indicator of animal welfare problems; thus, it is essential to rely on caregivers’ scores to rapidly identify such changes. 

We found that out of 24 raters, only 4 caretakers had several significant mean score differences among their tests. This result can have several interpretations. First, although no rater reported any misunderstandings or comments during the study, we believe that the discrepancies found in these four raters might be related to the fact that they did not fully understand the assignment’s instructions and/or the meaning of some of the adjectives on the questionnaire. Second, given that our study involved dolphinariums from three different countries, i.e., Spain, France, and Argentina, it might be that aspects associated with the cultural diversity and multiple lingual approaches could have affected the scores’ reliability. However, we think this is a very unlikely explanation here, as most of the significant mean score differences between tests 1 and 2 were only observed in four raters coming from two different centres, while there were other raters from the same centres that did not show such differences. In addition, previous studies have demonstrated that animal personality can be reliably assessed by raters from different cultural backgrounds and languages [21,28].

Animal caretakers and trainers should be familiar with the animals in their care as they observe them responding to a variety of situations daily. Raters can thus evaluate animals’ personality traits consistently based on long-term observations of dolphins because they should be able to recognise the regularity of certain behavioural traits as well as comprehend how they exhibit such traits [2,9]. However, our findings here should also be interpreted with a certain caution as it may be that consistencies of scores between raters from each centre could also be explained by preconceptions of the caretakers on the animals’ personalities. For example, caretakers could have potentially shared and discussed their own insight concerning the animals’ personalities and therefore reached certain conclusions about their personalities, even before they were asked to rate the dolphins. However, even if there were previous beliefs about the personality of each dolphin shared among the caretakers, given the questionnaire’s structure (i.e., it included 25 adjectives that were assessed for each individual on a seven-point scale), it would have been difficult to observe a good degree of agreement. We believe further research on the relationship between preconceptions about personality, caretakers scores, and data from behavioural observations to assess personality in bottlenose dolphins in captivity is interesting for the research community.

A limitation of using caretakers or trainers to score personality traits in bottlenose dolphins is that certain caretakers might not have spent much time observing their animals outside the training sessions. Unfortunately, this likely implies that their assessments could have been of low quality, i.e., showing not strong dependencies between observations within the same rater, or showing significant mean scores differences between tests 1 and 2, or only limited to the animals’ behaviours displayed during the controlled environment of a training session. Another explanation for the inconsistencies that some of the raters presented could be that these four raters did not know the dolphins that they worked with very well or that they had spent less time working with the animals compared with the other raters. Unfortunately, we do not have information about the experience of each caretaker, as our only requirement to fill in the questionnaire was that each person had worked closely with each to-be-assessed animal for at least 1 year, following the recommendations of personality studies with dolphins [23,24]. It would have been interesting, and is recommended for future research studies, to include rater experience and gender as factors in the statistical analysis [9,18].

Another interesting finding here was that it seemed that raters were more inconsistent in their scores between tests 1 and 2 when assessing female dolphins rather than males. An interesting point here would be to investigate why there was a significantly higher number of mean score differences between tests 1 and 2 in the caretakers’ scores when assessing females compared to males. It would be further interesting (and helpful) to know whether such significant differences might be somehow related to trainers’ human stereotypes in the sense that they give different meanings to adjectives, e.g., gentle, timid, or cooperative, depending on whether they are evaluating a male or a female. Another explanation could be that, perhaps, the current questionnaire includes adjectives that are generally easier evaluated in males rather than females, e.g., dominant, aggressive. A future study could be focused on investigating whether females’ and males’ personalities should actually be evaluated using different adjectives that best capture the overall traits of each of the personalities. We also believe a more detailed analysis should be elaborated to determine if the techniques for animals’ personality evaluation, e.g., questionnaires and adjectives, that we are using so far capture particular traits of males and females in the same manner, or, contrary, if they are biased towards, e.g., males, ignoring some adjectives which might be especially interesting for females. 

Finally, we observed that the trait ‘gentle’ was generally more challenging to assess compared to other traits as it showed the largest number of mean score differences between tests 1 and 2 among all scored adjectives. Although each adjective was properly described in the questionnaire to help raters understand them, the descriptions might not have been clear enough for all raters, which might be the case here for the adjective ‘gentle’. A future study could use an improved version of the questionnaire with more concise and thoroughly adjectives to reduce rater-related difficulties, e.g., difficulties understanding questions and adjectives, and re-formulate some of the current adjectives that showed discrepancies systematically, such as ‘gentle’.

## 5. Conclusions

In the current paper, we aimed to investigate the reliability of scores given by caretakers to evaluate the personality of bottlenose dolphins in captivity appropriately. To this end, we considered a two-step procedure to estimate, first, the degree of agreement in scores within each rater and across raters from each dolphinarium based on a variety of personality traits, as well as to identify the raters and centres showing significant mean score differences systematically. 

In our analysis, we used the intra-rater and intra-centre correlation coefficients to measure the degrees of agreement between the scores provided by the same rater in tests 1 and 2 and between the scores of raters from the same centre, respectively, as our first step for evaluating raters’ and centres’ reliabilities. In particular, we found good and fair intra-rater and intra-centre reliabilities, respectively, according to Chicchetti [27]. Therefore, it seemed that most of the caretakers scored animals in tests 1 and 2 consistently. Additionally, caretakers belonging to the same centres gave the animals rather comparable scores for each personality trait. In a second step, we identified the raters and centres who showed significant mean score differences, thus reducing the degrees of agreement. This last point is especially relevant here as detecting caregivers who have difficulty recognising the personalities of the animals in their care is critical, among other things, to prevent bad management decisions (e.g., developing better environmental enrichment programmes or identifying compatible individuals to create a socially stable group), and contribute to the improvement of each animal’s welfare in captivity.

## Figures and Tables

**Table 1:** List of adjectives and their meanings considered for each of the three sections included in the personality questionnaire (based on Highfill et al. [23] and Kuczaj et al. [24]). The terms in bold will be used as the baseline category.

Section	Adjectives	Definition
1: Interactions with the physical world	Not curious/**Curious**	Appears to be interested in new situations or objects.
	Not confident/**Confident**	Exhibits care in its actions; sure of itself.
	Not observant/**Observant**	Ready, attentive, watchful; appears to pay attention to surroundings.
	Not playful/**Playful**	Engages in play behaviour.
	Not creative/**Creative**	Approaches situations and addresses problems in novel, creative ways (e.g., finds various ways to play with a toy).
	Lethargic/**Energetic**	Animal is energetic if it moves around a lot. Locomotion can include swimming, leaping, etc.
	**Timid**/Bold	Animal is timid if it is hesitant, apprehensive, tentative.
2: Interactions with other dolphins	Not playful/**Playful**	Engages in play behaviour.
	Not observant/**Observant**	Ready, attentive, watchful; appears to pay attention to surroundings.
	Not tolerant/**Tolerant**	Inclined to be relaxed, easy-going, willing to adapt or change.
	Solitary/**Gregarious**	Animal is gregarious if it is agreeable and sociable. Appears to like the company of others. Seeks out social contact with other dolphins.
	Rough/**Gentle**	Animal is gentle if it is friendly and amicable towards other dolphins. Responds to others in an easy, kind manner. Not hostile. Not antagonistic.
	Not curious/**Curious**	Appears to be interested in interacting with dolphins and in new situations.
	Submissive/**Dominant**	Animal is dominant if it monitors its actions and exhibits a willingness to control, command.
	Not confident/**Confident**	Exhibits care in its actions; sure of itself.
	Not aggressive/**Aggressive**	Threatens or causes harm; high frequency of pushing, biting, or hitting other dolphins.
	**Timid**/Bold	Animal is timid if it is hesitant, apprehensive, tentative.
	Not cooperative/**Cooperative**	Cooperates with other dolphins to do a task. Not defiant.
3: Interactions with humans	Rough/**Gentle**	Animal is gentle if it is friendly and amicable towards humans. Responds to humans in an easy, kind manner. Not hostile. Not antagonistic.
	Not cooperative/**Cooperative**	Obeys; cooperates with instructions. Not defiant.
	Not observant/**Observant**	Ready, attentive, watchful; appears to pay attention to surroundings.
	Not playful/**Playful**	Engages in play behaviour.
	Not curious/**Curious**	Appears to be interested in interacting with humans and in new situations.
	Not aggressive/**Aggressive**	Threatens or causes harm; high frequency of pushing, biting, or hitting humans.
	**Timid**/Bold	Animal is timid if it is hesitant, apprehensive, tentative.

**Table 2:** Description of the six groups of dolphins that participated in the current study.

Group	1	2	3	4	5	6	Total
Centre	A	B	C	D	E	F	5
Number of dolphins	7F + 1M	6M	5F + 4M	3F + 2M	3F + 3M	6F + 2M	42
ID Raters	1–4	5–7	8–11	12–16	16–19	20–24	24

F = females, M = males.

**Table 3:** Statistically significant (5% of significance level) mean differences of scores in tests 1 and 2 for raters by section and adjective (in bold). Note that each rater always scored the same sample of dolphins.

Rater 7	Rater 8	Rater 10	Rater 12	Rater 13	Rater 16	Rater 18	Rater 21
**Gentle**, section 3 (−1.333) *p*-value = 0.037	**Playful**, section 2 (−1.555) *p*-value = 0.003	**Confident**, section 1 (−1.140) *p*-value = 0.041	**Gentle**, section 2 (−2.200) *p*-value = 0.002	**Observant**, section 1 (2.000) *p*-value = 0.004	**Tolerant**, section 2 (−1.703) *p*-value = 0.003	**Gentle**, section 3 (−1.333) *p*-value = 0.037	**Timid**, section 2 (−1.250) *p*-value = 0.024
	**Gentle**, section 2 (−2.333) *p*-value < 0.0001	**Creative**, section 1 (−1.169) *p*-value = 0.036	**Timid**, section 2 (−1.600) *p*-value = 0.022	**Playful**, section 1 (2.800) *p*-value = 0.0001			
	**Solitary**, section 2 (−1.222) *p*-value = 0.019	**Curious**, section 1 (−1.333) *p*-value = 0.011	**Aggressive**, section 3 (3.000) *p*-value < 0.0001	**Cooperative**, section 2 (1.400) *p*-value = 0.046			
	**Timid**, section 2 (−1.111) *p*-value = 0.033	**Observant**, section 1 (−1.458) *p*-value = 0.009	**Gentle**, section 3 (−2.400) *p*-value = 0.001	**Curious**, section 2 (3.600) *p*-value < 0.0001			
	**Tolerant**, section 2, (−2.000) *p*-value = 0.0001	**Timid**, section 1 (−1.507) *p*-value = 0.007		**Playful**, section 2 (4.400) *p*-value < 0.0001			
	**Aggressive**, section 3 (−1.770) *p*-value = 0.001	**Aggressive**, section 2 (−1.714) *p*-value = 0.004		**Solitary**, section 2 (−1.400) *p*-value = 0.046			
	**Gentle**, section 3 (−2.000) *p*-value = 0.0001			**Timid**, section 2 (1.800) *p*-value = 0.010			
				**Playful**, section 3 (3.400) *p*-value < 0.0001			

**Table 4:** Statistically significant (5% significance level) mean differences in scores between tests 1 and 2 for centre, section, and adjective (in bold).

Centre B	Centre C	Centre D	Centre E	Centre F
**Gentle**, section 3 (−1.333) *p*-value = 0.037	**Confident**, section 1 (−1.140) *p*-value = 0.041	**Observant**, section 1 (2.000) *p*-value = 0.004	**Tolerant**, section 2 (−1.703) *p*-value = 0.003	**Timid**, section 2 (−1.250) *p*-value = 0.024
	**Creative**, section 1 (−1.169) *p*-value = 0.036	**Playful**, section 1 (2.800) *p*-value = 0.0001	**Gentle**, section 3 (−1.333) *p*-value = 0.037	
	**Curious**, section 1 (−1.333) *p*-value = 0.011	**Gentle**, section 2 (−2.200) *p*-value = 0.002		
	**Observant**, section 1 (−1.458) *p*-value = 0.009	**Timid**, section 2 (−1.600) *p*-value = 0.022		
	**Timid**, section 1 (−1.507) *p*-value = 0.007	**Cooperative**, section 2 (1.400) *p*-value = 0.046		
	**Aggressive**, section 2 (−1714) *p*-value = 0.004	**Curious**, section 2 (3.600) *p*-value < 0.0001		
	**Playful**, section 2 (−1.555) *p*-value = 0.003	**Playful**, section 2 (4.400) *p*-value < 0.0001		
	**Gentle**, section 2 (−2.333) *p*-value < 0.0001	**Solitary**, section 2 (−1.400) *p*-value = 0.046		
	**Solitary**, section 2 (−1.222) *p*-value = 0.019	**Timid**, section 2 (1.800) *p*-value = 0.010		
	**Timid**, section 2 (−1.111) *p*-value = 0.033	**Tolerant**, section 2 (−1.703) *p*-value = 0.003		
	**Tolerant**, section 2, (−2.000) *p*-value = 0.0001	**Aggressive**, section 3 (3.000) *p*-value < 0.0001		
	**Aggressive**, section 3 (3.000) *p*-value < 0.0001	**Gentle**, section 3 (−2.400) *p*-value = 0.001		
	**Gentle**, section 3 (−2.400) *p*-value = 0.001	**Playful**, section 3 (3.400) *p*-value < 0.0001		

**Table 5:** Statistically significant (5% significance level) mean differences in scores between tests 1 and 2 for rater, centre, adjective (in bold), and sex of the dolphin. Female results in black, male results in blue.

Rater 8	Rater 10	Rater 12	Rater 13	Rater 16	Centre C	Centre D
**Playful**, section 2 (−1.600) *p*-value = 0.025	**Confident**, section 1 (−1.537) *p*-value = 0.043	**Gentle**, section 2 (−2.667) *p*-value = 0.004	**Curious**, section 1 (2.667) *p*-value = 0.004	**Tolerant**, section 2 (−1.500) *p*-value = 0.022	**Confident**, section 1 (−1.537) *p*-value = 0.043	**Curious**, section 1 (2.667) *p*-value = 0.004
**Gentle**, section 2 (−2.400) *p*-value = 0.001	**Aggressive**, section 2 (−1.750) *p*-value = 0.029	**Aggressive**, section 3 (3.000) *p*-value = 0.001	**Playful**, section 1 (3.667) *p*-value = 0.0001		**Aggressive**, section 2 (−1.750) *p*-value = 0.029	**Playful**, section 1 (3.667) *p*-value = 0.0001
**Tolerant**, section 2, (−2.400) *p*-value = 0.001	**Observant,** section 1 (−2.058) *p* -value = 0.017	**Gentle**, section 3 (−2.333) *p*-value = 0.012	**Curious**, section 2 (3.600) *p*-value < 0.0001		**Playful**, section 2 (−1.600) *p*-value = 0.025	**Curious**, section 2 (3.600) *p*-value < 0.0001
**Aggressive**, section 3 (−1.800) *p*-value = 0.012	**Timid,** section 1 (−2.055) *p* -value = 0.018	**Aggressive,** section 3 (3.000) *p* -value = 0.008	**Curious**, section 2 (3.333) *p*-value = 0.0003		**Gentle**, section 2 (−2.400) *p*-value = 0.001	**Curious**, section 2 (3.333) *p*-value = 0.0003
**Gentle**, section 3 (−2.000) *p*-value = 0.005		**Gentle,** section 3 (−2.500) *p* -value = 0.027	**Playful**, section 2 (4.000) *p*-value < 0.0001		**Tolerant**, section 2, (−2.400) *p*-value = 0.001	**Playful**, section 2 (4.000) *p*-value < 0.0001
**Gentle** , section 2 (−2.250) *p* -value = 0.005			**Timid**, section 2 (3.000) *p*-value = 0.001		**Aggressive**, section 3 (−1.800) *p*-value = 0.012	**Timid**, section 2 (3.000) *p*-value = 0.001
**Aggressive** , section 3 (−1.750) *p* -value = 0.029			**Playful**, section 3 (3.667) *p*-value = 0.0001		**Gentle**, section 3 (−2.000) *p*-value = 0.005	**Gentle**, section 2 (−2.667) *p*-value = 0.004
**Gentle** , section 3 (−2.000) *p* -value = 0.012			**Observant** , section 1 (3.000) *p* -value = 0.008		**Observant** , section 1 (−2.058) *p* -value = 0.017	**Tolerant**, section 2 (−1.500) *p*-value = 0.022
			**Cooperative** , section 2 (3.500) *p* -value = 0.002		**Timid** , section 1 (−2.055) *p* -value = 0.018	**Observant** , section 1 (3.000) *p* -value = 0.008
			**Curious** , section 2 (4.000) *p* -value = 0.0004		**Gentle** , section 2 (−2.250) *p* -value = 0.005	**Cooperative** , section 2 (3.500) *p* -value = 0.002
			**Playful** , section 2 (5.000) *p* -value < 0.0001		**Aggressive** , section 3 (−1.750) *p* -value = 0.029	**Curious** , section 2 (4.000) *p* -value = 0.0004
			**Playful** , section 3 (3.000) *p* -value = 0.008		**Gentle** , section 3 (−2.000) *p* -value = 0.012	**Playful** , section 2 (5.000) *p* -value < 0.0001
						**Playful** , section 3 (3.000) *p* -value = 0.008
						**Aggressive** , section 3 (3.000) *p* -value = 0.008
						**Gentle** , section 3 (−2.500) *p* -value = 0.027

## Data Availability

The data presented in this study are available upon reasonable request.
